# Temporizin and Temporizin-1 Peptides as Novel Candidates for Eliminating *Trypanosoma cruzi*

**DOI:** 10.1371/journal.pone.0157673

**Published:** 2016-07-06

**Authors:** André L. A. Souza, Robson X. Faria, Kátia S. Calabrese, Daiane J. Hardoim, Noemi Taniwaki, Luiz A. Alves, Salvatore G. De Simone

**Affiliations:** 1 FIOCRUZ, Center for Technological Development in Health (CDTS)/National Institute of Science and Technology for Innovation on Neglected Diseases (INCT-IDN), Rio de Janeiro, RJ, Brazil; 2 FIOCRUZ, Oswaldo Cruz Institute, Laboratory of Experimental and Computational Biochemistry of Pharmaceuticals, Rio de Janeiro, RJ, Brazil; 3 FIOCRUZ, Oswaldo Cruz Institute, Laboratory of Toxoplasmosis and other Protozoosis, Rio de Janeiro, RJ, Brazil; 4 FIOCRUZ, Oswaldo Cruz Institute, Laboratory of Imunomodulation and Protozoology, Rio de Janeiro, RJ, Brazil; 5 FIOCRUZ, Adolfo Lutz Institute, Electronic Microscopy section, Araçatuba, São Paulo, SP, Brazil; 6 FIOCRUZ, Oswaldo Cruz Institute, Laboratory of Cellular Communication, Rio de Janeiro, RJ, Brazil; 7 Federal Fluminense University, Biology Institute, Department of Cellular and Molecular Biology, Niterói, RJ, Brazil; Nanyang Technological University, SINGAPORE

## Abstract

Tropical diseases caused by parasitic infections continue to cause socioeconomic distress worldwide. Among these, Chagas disease has become a great concern because of globalization. Caused by *Trypanosoma cruzi*, there is an increasing need to discover new, more effective methods to manage infections that minimize disease onset. Antimicrobial peptides represent a possible solution to this challenge. As effector molecules of the innate immune response against pathogens, they are the first line of defense found in all multi-cellular organisms. In amphibians, temporins are a large family of antimicrobial peptides found in skin secretions. Their functional roles and modes of action present unique properties that indicate possible candidates for therapeutic applications. Here, we investigated the trypanocide activity of temporizin and temporizin-1. Temporizin is an artificial, hybrid peptide containing the N-terminal region of temporin A, the pore-forming region of gramicidin and a C-terminus consisting of alternating leucine and lysine. Temporizin-1 is a modification of temporizin with a reduction in the region responsible for insertion into membranes. Their activities were evaluated in a cell permeabilization assay by flow cytometry, an LDH release assay, electron microscopy, an MTT assay and patch clamp experiments. Both temporizin and temporizin-1 demonstrated toxicity against *T*. *cruzi* with temporizin displaying slightly more potency. At concentrations up to 100 μg/ ml, both peptides exhibited low toxicity in J774 cells, a macrophage lineage cell line, and no toxicity was observed in mouse primary peritoneal macrophages. In contrast, the peptides showed some toxicity in rat adenoma GH3 cells and Jurkat human lymphoma cells with temporizin-1 displaying lower toxicity. In summary, a shortened form of the hybrid temporizin peptide, temporizin-1, was efficient at killing *T*. *cruzi* and it has low toxicity in wild-type mammalian cells. These data suggest that temporizin-1 might be a candidate for Chagas disease therapy.

## Introduction

*Trypanosoma cruzi* is an intracellular protozoan parasite that is responsible for Chagas disease. This disease is endemic throughout regions of Mexico and Latin America, where 11 million people are infected and 25 million are at risk [1]. This disease is transmitted to humans along with domestic and wild animals primarily by large, blood-sucking reduviid insects of the subfamily Triatominae [2]. Chagas disease can also be caused by blood transfusion and vertically from mother to infant. Approximately 30% of infected people develop debilitating or life-threatening medical conditions [3], namely heart arrhythmias, megaesophagus and megacolon.

The initial, acute phase of a *T cruzi* infection lasts for 4–8 weeks and transitions into the chronic phase for the lifespan of the host [4, 5]. Symptoms will appear 1–2 weeks after an individual is exposed to an infected triatomine insect. When exposure occurs through transfusion with infected blood, symptoms can take up to a few months to appear. Often, the initial phase is asymptomatic or might be present as a self-limiting febrile illness. In general, clinical manifestations that occur during this phase resolve spontaneously in 90% of infected patients, and 60–70% of them will never develop a clinically apparent disease. The remaining 30–40% of patients will subsequently develop a determinate form of chronic disease (cardiac, digestive or cardiodigestive) that can present several decades after infection.

Anti-trypanosomal treatment is recommended for all acute, congenital infections in children, reactivated infection cases and patients of up to 18 years of age with the chronic disease [6, 7]. Although there is currently no convincing therapeutic strategy for Chagas disease, it is treated with benznidazole and nifurtimox [8, 9], even though these drugs are very toxic [10–12]. Generally speaking, the control of this disease depends on prophylaxis and therapeutic anti-parasite drugs [13]. However, the inappropriate use of these drugs has led to an increase in parasite resistance, as for example, in African trypanosomes [14, 15]. Taken together, this information indicates an urgent need for novel agents to cure infections and prevent disease.

In keeping with this idea, anti-microbial peptides (AMPs) are efficient molecules that have functioned as a defense mechanism throughout evolution [16]. They participate in the innate immune systems of animals [17] with a broad spectrum of activity against plants, bacteria, fungi, parasites and viruses [18–26]. These peptides can attack pathogens by interfering with intercellular cell function, affecting the membrane potential of microorganisms or by forming pores in plasma membrane. In all cases, the result is cell death through necrosis or apoptosis. In addition, they may exhibit diverse effector functions that modulate the host innate immune responses [18, 27].

Recent studies have shown that AMPs are used as antibiotic substances found in the cellular secretions produced by fungi and bacteria. In general, antimicrobial peptides are generated from within the pre-propeptide region of proteins. Some peptides originate from the N-terminal signal sequences of proteins that are synthesized in the endoplasmic reticulum, while others are contained in segments that are conserved in the C-terminal sequences of protein hormones and enzymes [28]. AMPs have been grouped on the basis of their primary structure, size and length. Among life forms, the highest producers, and the largest source of antimicrobial peptides, are frogs [29, 30]. Of these peptides, the temporin family represents a large group of peptides with therapeutically desired characteristics of lytic activity against various microorganisms and low toxicity against mammalian cells.

The refractory tendency of frogs to *Trypanosoma cruzi* [31] and the diversity of antimicrobial peptides they produce [32–35] has guided our laboratory to synthesize a hybrid peptide molecule composed of regions from temporin A and gramicidin with a poly-leucine/lysine carboxy terminus. Named temporizin, our objective was to test the biocidal action of this peptide against *T*. *cruzi* and to assess its toxicity in mammalian cells. Assays with 3-(4,5-dimethylthiazol-2-yl)-2,5-diphenyltetrazolium bromide (MTT) and propidium iodide (PI) uptake demonstrated its toxicity toward T. cruzi that was confirmed by the cellular destruction observed in treated parasites by electron microscopy (EM). An LDH assay revealed its low toxicity towards mammalian cells. Temporizin-1 was engineered to further minimize toxicity against mammalian cells while maintaining toxicity against parasites. The observed reduction in toxicity observed for temporizin-1 may be due to its formation of ionic channels in mammalian cells membrane with unitary conductance inferior to that measured for temporizin peptide.

## Material and Methods

### Reagents

All reagents were purchased from Sigma Chemical (St. Louis, MO, U.S.A.).

### Cell culture

Mouse peritoneal macrophages were harvested through the peritoneal lavage. Male Swiss Webster were euthanized in a chamber CO_2._ In sequence, we applied 10 mL of DMEM medium to harvest peritoneal cells. Cells were collected by centrifugation and, after suspension in fresh media, aliquots (0.5 ml) were placed in microplate wells for a 30 min incubation at 37°C in a humidified atmosphere of 5% CO_2_. Non-adherent cells were removed by washing with medium and the remaining, adherent cells were harvested into phenol red free DMEM with 10% fetal bovine serum (FBS) and gentamicin (1 μl /ml) for use in subsequent experimental procedures [36]. All animal manipulations adhered to the Ethical Principles in Animal Experimentation as adopted by the Brazilian College of Animal Experimentation and specific protocols were approved by the Fiocruz Research Ethics Committee (number LW-033/12).

The cell lines J774, GH3 and Jurkat were cultured in RPMI 1640 medium containing 10% fetal calf serum, penicillin (100 U/ml) and streptomycin (100 μg/ml) in 35-mm Petri dishes for two to five days. Cells were incubated at 37°C in a humidified, 5% CO_2_ atmosphere for the all experiments.

### Parasite culture and anti-parasitic assays

The epimastigote forms of the *T*. *cruzi* strain Y were maintained in liver infusion tryptose (LIT) medium. Parasites in the log phase (5^th^ day of culture) were harvested and washed three times in PBS by centrifugation (1500 g x 30 min, 25°C). The parasites in the last pellet were suspended in PBS and immediately disrupted by 10 cycles of freeze and thaw before centrifugation (100,000 g x 45 min, 4°C). The pellet and supernatant were separated collected.

### Antimicrobial peptide temporizin and peptide synthesis

The structure of the temporizin was engineered based on three peptides described in the literature, although the antimicrobial activity of temporin on trypanosomatids was not reported. We began with an N-terminal structure similar to that of temporin, which consists of peptides that are found in the secretions of the frog *Rana temporaria* [37], an animal naturally refractory to *Trypanosoma cruzi* [31]. In the central region of the peptide, from the leucine at position 3 to the tryptophan at position 12, we constructed a structure similar to that found in gramicidin A guided by the portion that interacts with biological membranes and is responsible for its pore forming characteristic [38–41]. The C-terminus was designed with alternating leucine and lysine amino acids based on the reported evidence for its contribution to peptide stability and its potential affinity for polysaccharide surface structures found in microorganisms. In addition, some reports show that arginine residues confer greater stability to peptide fragments as well as lower toxicity [42]. A comparison of the temporin, gramicidin, poly-Leu/Lys and temporizin sequences is shown in Table 1. Temporizin and temporizin-1 were produced by solid phase synthesis using the F-moc strategy in an automatic synthesizer (PSS-8—Shimadzu, Japan) using reagents prequalified for purity by high performance liquid chromatography (HPLC) with a reverse-phase column. The sequence of the peptide products was confirmed by mass spectrometry.

**Table 1:** The amino acid sequences of temporin A, gramicidin, poly-leu, temporizin and temporizin-1 peptides. The entire sequences of the peptides are shown. The temporizin peptide was formed from the junction of the N-terminal temporizin A with the portion of the gramicidin pore-forming section and the C-terminus of the poly-leu peptide. Temporizin-1 has a reduction in the portion responsible for gramicidin pore formation.

Peptide	Source	Linear Sequency	Charge in Neutral PH
**Temporizin**	**LaBECFAR**	**FLPLWLWLWLWLWKLK**	**+2**
**Temporin A**	***Rana temporaria***	**FLPLIGRVSLSGIL–NH2**	**+2**
**Temporin B**	***Rana temporaria***	**FLPLIGRVLSSLL–NH**	**+2**
**Temporin F**	***Rana temporaria***	**FLPLIGKVLSGIL–NH2**	**+2**
**Gramicidin A**	***Bacillus brevis***	**HCO-VGALAVVVWLWLWLW-NHCH20H**	**0**
**Poly-Leu**	**Sarcophaga peregrina**	**KLKLLLLLKLK**	**+4**
**Temporizin 1**	**LaBECFAR**	**FLPLWLWLWRKLK**	**+3**

### Measuring the temporizin concentration

The temporizin concentration was determined by measuring the optical density at 205 nm with a molar extinction coefficient that was previously determined for peptides of 12–15 residues. Alternatively, the concentration was determined by an automatic amino acid analyzer (Shimadzu) in collaboration with Dr. Philip Quirino (INCQS-FIOCRUZ) or by amino acid analysis with an HPLC system (Shimadzu, model 6A) followed by OPA derivatization (emission 450 nm excitation and 350 nm).

### Mass spectrometry

Electronspray mass spectrometry was performed on a Q-TOF Ultima API instrument (Micromass). Temporizin samples were analyzed in positive mode after injection into a manual rheodyne injector and then carried by an LC-10AD Shimadzu pump at 20 μL/min under constant flow in a solution of 50% acetonitrile and 0.5% formic acid. Equipment control and data acquisition was performed with MassLynx v 4.0 software. The deconvolution spectra were generated by a module of MaxEnt I software.

For the 'de novo' sequencing of temporizin, ions of interest were selected within a mass window of ± 0.5 Da and fragmented by a collision induced by argon. The spectrum of fragments was handled by a MaxEnt module III and then analyzed with a BioLynx sequencing module for 'new' peptides.

Mass spectrometry was performed on a MALDI-ToF/Pro instrument (Amersham). Temporizin samples in solution were mixed 1:1 (v:v) with a supersaturated matrix solution for peptides (cinnamic acid) or a matrix for proteins (sinapic acid), deposited on the plate sample (0.4 to 0.8 ml) and air-dried. Automatic equipment control and data acquisition were provided by manufacturer software.

### Lytic assay with erythrocytes

The hemolytic activity of temporizin was analyzed in a 1% suspension (in a total volume of 1000 μL) of human erythrocytes (1×10^7^) previously washed with PBS (pH 7.4). Cells were incubated at 37°C for 30 min in a range of temporizin concentrations (0.1 to 1000 μmol mL^-1^). After centrifugation (4000 x g, 5 min, 4°C), a 100 μL sample of the supernatant was collected, diluted to 1 ml with PBS and its absorbance was measured at 540 nm. The percent lysis was determined by comparing the optical density to that of a suspension treated with Triton X-100 (0.2%), which was defined as 100%.

### Analysis of the trypanocidal activity of temporizin through Propidium Iodide uptake by fluorescence microscopy and flow cytometry

The trypanocide activity of the peptides was evaluated by using treating *T*. *cruzi* epimastigotes (1.0 X 10^6^ ml^-1^) with 1 ng to 100 μg/ml peptide concentrations in a 30 or 60 min incubation at 28°C. After the incubation period, the culture was incubated an additional 10 min in the presence of PI (0.05 μg/mL). For fluorescence microscopy, representative images were captured by a Nikon digital camera on a TE-2000S inverted microscope (Nikon) and analyzed with Image J software. For flow cytometry, cells were applied to a FACS Calibur and data acquired by the Cell Quest software. Data was analyzed with the *WinMDI* program. Negative controls consisted of treatment with saline only or formol (0.3 mg/ml or 3 mg/ml) while positive controls were treated with a range of Triton-X concentrations (0.0000001%–0.1%). In some flow cytometry experiments, viable trypanosomes were labeled with Calcein-AM and non-viable trypanosomes with PI.

### Whole cell Electrophysiological measurements

Patch clamp experiments were performed essentially as described elsewhere [43]. Whole-cell experiments were performed at 30–37°C after a high-resistance seal (1–10 GΩ) was established by gentle suction. Voltage clamp protocols were applied from holding potentials of -80 to 80 mV. Reversal potentials were calculated from the I-V curves. The whole-cell configuration was performed with a holding potential of -60mV. The currents were recorded for the Axopatch-1D amplifier (Axon Instruments, San Mateo, U.S.A) and filtered with a corner frequency of 5 kHz (8-pole Bessel filter), digitized at 20–50 kHz with a Digidata 1320 interface (Axon Instruments, Palo Alto, CA, U.S.A) and acquired on a personal computer with an Axoscope and pCLAMP 9.0 software (Axon Instruments, Palo Alto, CA, U.S.A).The series resistance was 8–12 MΩ for all experiments in saline solution A, and no compensation was applied for currents <400 pA due to the absence of leakage. Above this level, the currents were compensated by 82%. The cell capacitance (24 ± 3.9 pF, n = 68) was measured by applying a 20 mV hyperpolarizing pulse from a holding potential of -20 mV; the capacitive transient was then integrated and divided by the amplitude of the voltage step (20 mV). The recordings were accepted if the current and membrane conductance returned to within 1–5% of the control values after application of agonist indicating that the large conductance increases were not related to cell lysis from the loss of the seal. Experiments in which the series resistance increased substantially during the measurement were discarded. The drug application was performed as previously described [36] under perfusion (RC-24 chamber, Warner Instrument Corporation, Handem, USA). All drugs were dissolved in saline solution immediately before use. The ion currents were studied by a single application of agonist from 5 s to 30 s.

### Membrane potential control in cell-attached configuration

Solution A was used inside the pipette and solution B was used in the bath to record single-channel currents. The rationale for this procedure in the cell-attached configuration was two-fold as follows: 1) the solution A inside the pipette led to more physiological ion gradients across the patch, and thus we could study the ionomycin-activated phenomena more efficiently; and 2) the solution C in the bath should completely depolarize the cell membrane (~0 mV), and thus the real value of the voltage potential across the patch was the applied nominal holding potential. This setup circumvented the inconvenience of having to measure the cell potential to calculate the real holding potential.

### Saline solutions for electrophysiology

Different saline solutions were employed in the pipette or in the bath depending on the protocol as follows: **solution SE** (in mM): 150 NaCl, 5 KCl, 1 MgCl_2,_ 1 CaCl_2_ and 10 HEPES, pH 7.4; **solution SI** (in mM): 150 KCl, 5 NaCl, 1 MgCl_2_, 1 CaCl_2_, 10 HEPES and 0.1 EGTA, pH 7.4.

### LDH Release Assay

The presence of LDH in the media was detected in all experiments by using a cytotoxicity detection kit (Sigma kit for LDH) according to the manufacturer’s instructions and compared with the total LDH quantity in the cells. Cell supernatants were tested for LDH, a reducer of NAD^+^, which then converts tetrazolium dye into a soluble, colored formazan derivative. The initial rate of absorbance was measured in a plate reader at 490 nm. We incubated the peptides within a range from 0.001 μg to 100 μg (depending on the peptide) for 1 hour in the presence of J774, GH3, Jurkat cells and peritoneal macrophages. benznidazole was used in the concentrations between 0,1 μM and 500 μM for 1 hour.

### MTT Assay

*T*. *cruzi* epimastigote cultures (strain Y) were grown at 28°C in Schneider’s and TC100 media containing 5% heat-inactivated FBS. The trypanocidal assay (5 h) was performed in the absence of FBS (see below). *T*. *cruzi* samples were harvested on days 4 and 5 of the culture, and the antiparasitic activity of the different peptides was determined by estimating cell viability by MTT method [44]. In brief, parasites were washed twice by centrifugation (1000 x g for 10 min) with PBS (DIFCO) and resuspended in fresh culture medium to a known cell concentration (4 x 10^7^ parasites/ml) for the assays involving 5 h of incubation with peptides. The parasite suspension (50 μl) was incubated in 96-well microplates at 28°C with an equal volume of serially diluted peptides or sterile Milli-Q water as controls for 5 h to evaluate the trypanocide activity. After this time, each well received 30 μl of MTT solution (4 mg/ml), and the reduced formazan was solubilized by adding 50 μl of isopropyl alcohol after 4 h to the *T*. *cruzi*. The parasite viability was determined spectrophotometrically at 540 nm. In positive controls, the parasites were previously killed with 0.01% Triton X-100 (30 min), or with the trypanocidal compound Rochagan (benznidazole, 100 μM) for *T*. *cruzi*. The results were normalized to those of the corresponding controls in the absence of the peptide, and the percentage of viable parasites was determined.

### Transmission Microscopy electron

Epimastigotes forms of *T*. *cruzi*, were treated with temporizin, temporizin 1 or gramicidin at a concentrations of 1μg/mL. At time points of 30 minutes, 60 minutes, 90 minutes and 3 hours, parasites were collected and fixed with 2.5% glutaraldehyde (Sigma, USA) in 0.1M sodium-cacodylate buffer (pH 7.2) for 4 hours. Next, parasites were washed three times with 0.1 M sodium-cacodylate buffer and post-fixed in a solution containing 1% osmium tetroxide, 0.8% ferrocyanide, and 5 mM calcium chloride followed by a wash in 0.1M sodium-cacodylate buffer. Parasites were then dehydrated in EM-grad acetone and embedded in epoxy resin. Ultrathin sections were cut and the sections stained with uranyl acetate and lead citrate. Samples were examined by a transmission electron microscope JEM-1011 (JEOL, Japan).

### Data analysis

We normalized the fluorescence data for the maximum fluorescence value with Microsoft Excel and plotted the results with GraphPad Prism version 3.0 (San Diego, CA, U.S.A). The PI uptake maximum was quantified following temporizin stimulation. The basal values were subtracted, and the curves were normalized against the maximum fluorescence intensity obtained for the 3 mg/ml formalin application. The data were expressed as the mean ± SD (standard deviation) as indicated in the text. The fluorescence intensity (photos) was measured with free Image J software (V1.40g; National Institutes of Health, Maryland, U.S.A.) or WinMDI software (cytometry). In order to test if the samples follow a Gaussian distribution, the D’ Agostino and Pearson (normality test) was used. If the data follow a Gaussian distribution, an appropriate parametric test was applied, if not; an appropriate non-parametric test was applied. The used tests were specified in the figure legends and were two-tailed paired. P values of 0.05 or less were considered significant.

## Results

### Temporizin effects on *T*. *cruzi* are concentration-dependent

**Fig 1:**
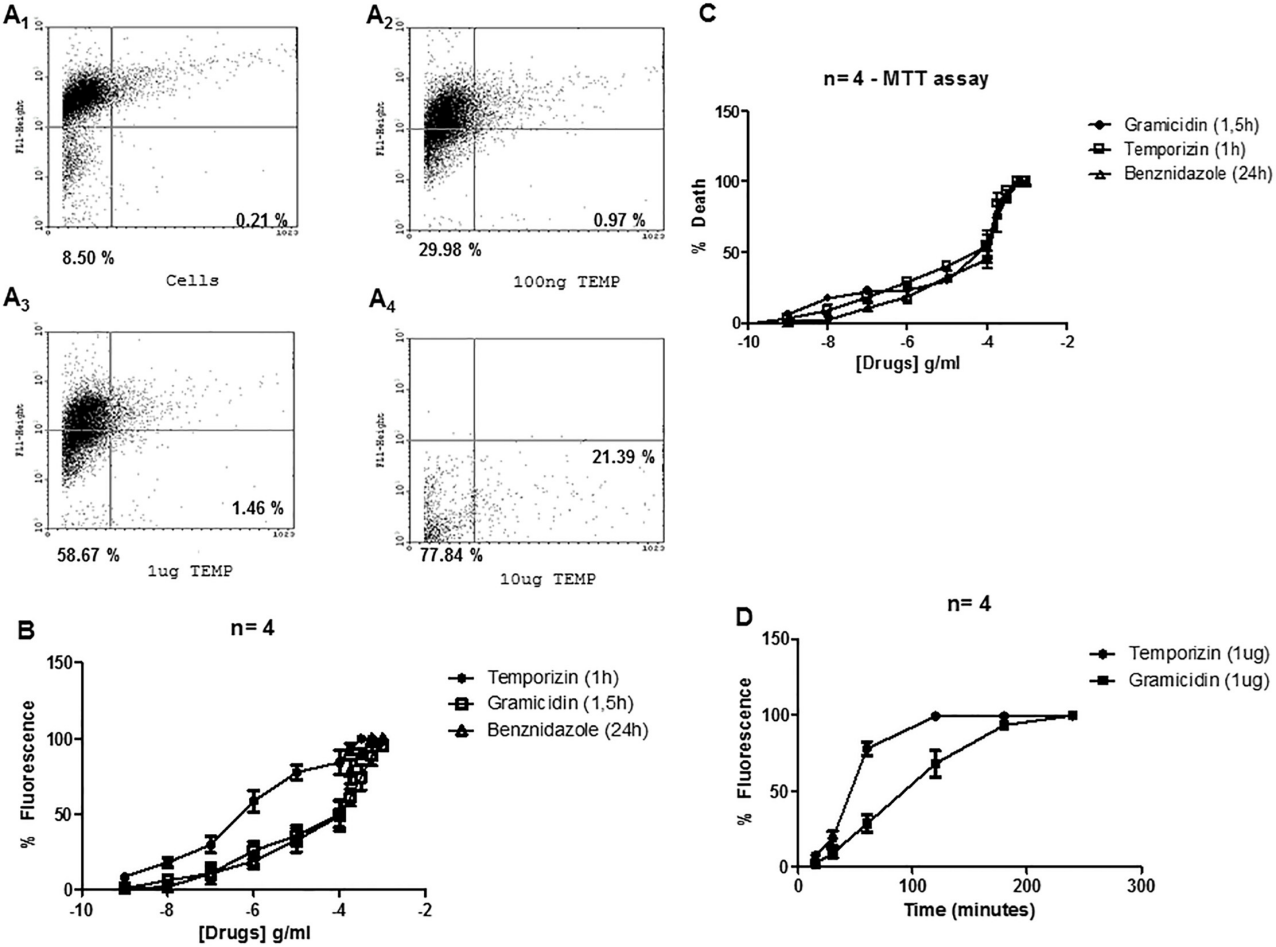
Temporizin kills *Trypanosoma cruzi*. A: T. cruzi were incubated, for the cell permeabilization assay, at 27°C for 45 minutes in the absence of treatments (A1) or presence of 100 ng/ml (A2), 1 μg/ml (A3) and 10 μg/ml temporizin (A4) for 60 minutes. B: Dose-response curves of temporizin, gramicidin and benznidazole acting against T. cruzi for 60, 90 and 1440 minutes, respectively. The trypanocide action was quantified for PI uptake using flow cytometry. C: MTT assays in the same conditions to B. D: Temporal quantification measuring PI entry after treatment with 1 μg/ml temporizin or gramicidin. These values represent the mean ± SD of three to five experiments performed on different days.

We tested temporizin concentrations ranging from 1 ng/mL to 100/mL μg to evaluate its trypanocide action by MTT assay and flow cytometry. In comparison, we used as a positive control gramicidin, a widely used peptide with toxicity against a diverse spectrum of wild-type mammalian and non-mammalian cell types. The temporizin pore is composed of the same amino acid sequence as that of gramicidin (see Material and Methods). Initially, trypanosomes were incubated for 60 min with 1 μM Calcein-AM followed in sequence by exposure to 1 μg/ml temporizin or Gramicidin for an additional 60 minutes. The efflux of calcein from trypanosomes was visualized by flow cytometry (Fig 1A).

In the plot of fluorescence versus forward scatter (FSC), the calcein fluorescence represents the majority of the signal in the superior quadrants containing trypanosomes with intact membrane. The inferior quadrants represent calcein efflux and consequent lesion on trypanosome membrane. The negative control (cells), we quantified an calcein fluorescent signal of 91.29% in the superior quadrants (viable trypanosomes) and a signal of 8.71% in the inferior quadrants, dead trypanosomes, as expected to no treated trypanosomes (Fig 1A_1_). Trypanosomes treated with the positive control Triton-X 100 showed an elevated calcein signal of 98% in the inferior quadrants (data not shown). To control for the peptide effect, medium was heated to 100°C prior to adding the temporizin peptide. As expected for the denatured peptide, no toxicity towards trypanosomes was observed (data not shown). In the Fig 1A_2-4_, we show a dose-dependent relation between the temporizin concentration and the percentage of dead parasite after treatments for 1 hour. This relationship measured by flow cytometry produced an EC_50_ value of 795 ng/ml, 150.4 μg/ml and 109.0 μM to temporizin, gramicidin and benzonidazole, respectively (Fig 1B). In an MTT assay, the EC_50_ values were 855. 7 ng/ml, 155 μg/ml and 122.8 μM for temporizin, gramicidin and benzonidazole, respectively (Fig 1C). Based on EC_50_ value, we evaluated the kinetic action of the peptides (Fig 1D). Temporizin (1 μg/ml) and gramicidin (150 μg/ ml) reached the maximal response in distinct times. Temporizin had a maximal effect in 2 hours and Gramicidin in 3 hours. We used submaximal times of 1 hours and 1.5 hours for subsequent experiments with temporizin and gramicidin, respectively.

### Partial toxicity mediated by Temporizin in mammalian cells

**Fig 2:**
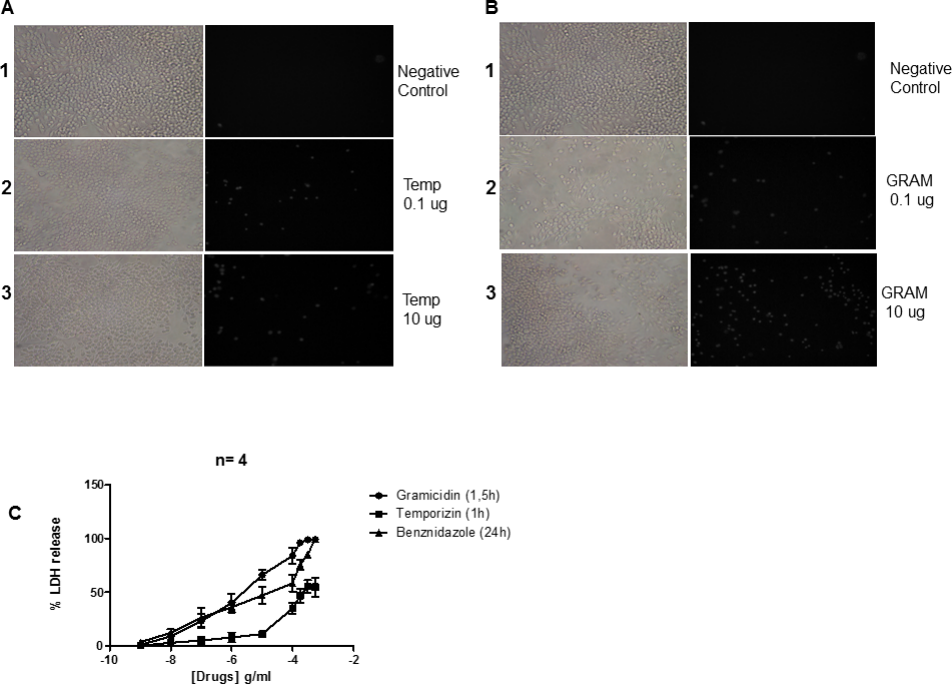
Temporizin causes moderate toxicity in mammalian J774 cells. J774 cell line was incubated at 37°C for 60 minutes in the presence of temporizin (0.1 and 10 μg/ml; Panel A) or gramicidin (0.1 and 10 μg/ml; Panel B), respectively. C: A quantification of the results obtained by PI entry assay after treating J774 cells with temporizin, gramicidin and benznidazole at different concentrations for 60, 90 and 1440 minutes. Gramicidin and temporizin toxicity was quantified by LDH Release Assay. The values represent the mean ± SD of four to six experiments (cell permeabilization assay) and three experiments (LDH release assay) performed on different days. *p<0.05, **p<0.01 compared with the corresponding negative control.

Several antimicrobial peptides can penetrate and pass through the plasma membrane of mammalian cells, which can have unwanted effects for a therapeutic drug. Therefore, we tested temporizin actions on cultured mammalian cells. The treatment with temporizin (0.1 and 10 μg/ml) for 1 hour caused membrane damage observed as PI entry with the mouse J774.G8 cell lineage (Fig 2A) in comparison to non-treated cells. Gramicidin treatment (1 and 100 μg/ml) for 1.5 hours was more harmful than temporizin (Fig 2B). The graph of the dose-response curve demonstrates the toxic action of both peptides, along with benzonidazole, as illustrated in (Fig 2C) that measured the extracellular LDH activity. The determined EC_50_ of temporizin was 116. 9 μg/ml. For gramicidin, it was 2. 9 μg/ml and benzonidazole had an EC_50_ of 83.16 uM. The temporizin EC_50_ value towards J774 cells was about 100-fold greater than the EC_50_ value calculated to kill trypanosomes.

### Temporizin-modified peptide kills *T*. *cruzi*

**Fig 3:**
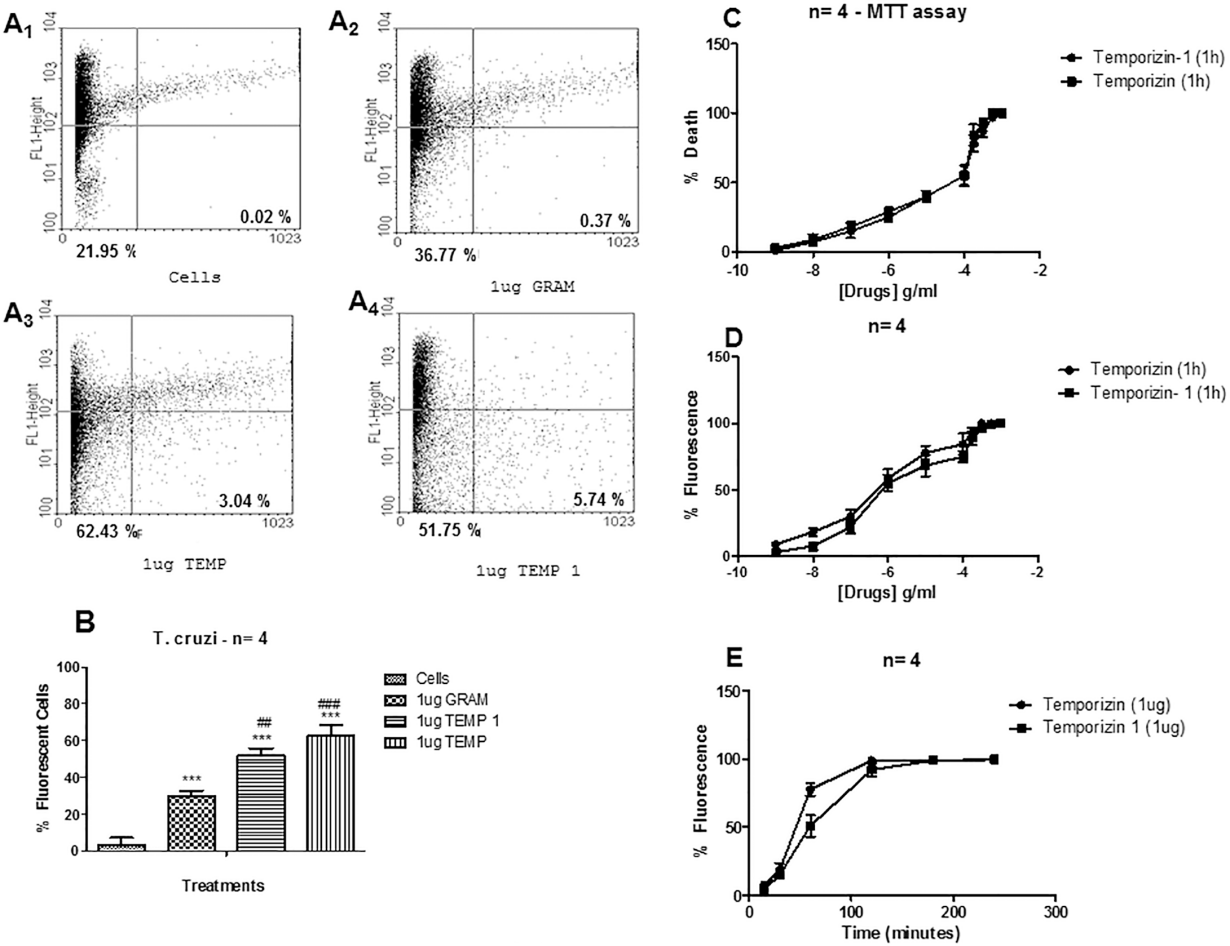
Temporizin-1 possesses toxicity against *T*. *cruzi*. A: *T*. *cruzi* was incubated at 37°C for 60 minutes for a flow cytometry assay. A single dose of 1 μg/ml was applied to compare the efficiency of the gramicidin (A2), temporizin A (A3) and temporizin-1 (A4) peptides in killing *T*. *cruzi*. B: A quantification of the results obtained in A. C: MTT assay using varied concentrations of temporizin and temporizin-1 for 60 minutes. D: PI entry quantification after the treatment with crescent temporizin and temporizin-1 doses for 60 minutes. E: Temporal assay after treatment with 1 μg/ml temporizin and temporizin-1. The values represent the mean ± SD of three to five experiments (flow cytometry and cell permeabilization assay) and three experiments (MTT assay) performed on different days. *p<0.05, **p<0.01 compared with the corresponding negative control.

Since the activity of the temporizin peptide demonstrated a toxic effect in wild-type mammalian cells, which would subsequently reduce its therapeutic value, we modified its linear sequence to obtain temporizin-1. The sequence of this new peptide is shown in Table 1. We compared the effect of temporizin-1 to temporizin on trypanosome viability. In a representative flow cytometry experiment, temporizin-1 killed 57.49% of the parasites, temporizin 65.47% and gramicidin 37.14% (Fig 3A_1-4_ and 3B). Cytotoxic assay measured by MTT and flow cytometry showed that temporizin-1 produce a trypanocidal activity similar to temporizin peptide (Fig 3C and 3D). The EC_50_ values determined for MTT assay was 887.2 ng/ml for temporizin- 1 and 849.3 ng/ml for temporizin (Fig 3C). The EC_50_ values to flow cytometry was 817.3 ng/ml to temporizin-1 and 795.1 ng/ml to temporizin (Fig 3D). The time course analysis exhibited similar temporal exponential association profile, but the time constant (t) to temporizin was 40.26 min compared to 65.57 minutes for temporizin-1 (Fig 3E). For subsequent experiments, we used a time of 1.5 hour for incubations with the temporizin-1 peptide.

### Temporizin-1 possesses low toxicity towards mammalian cells

**Fig 4:**
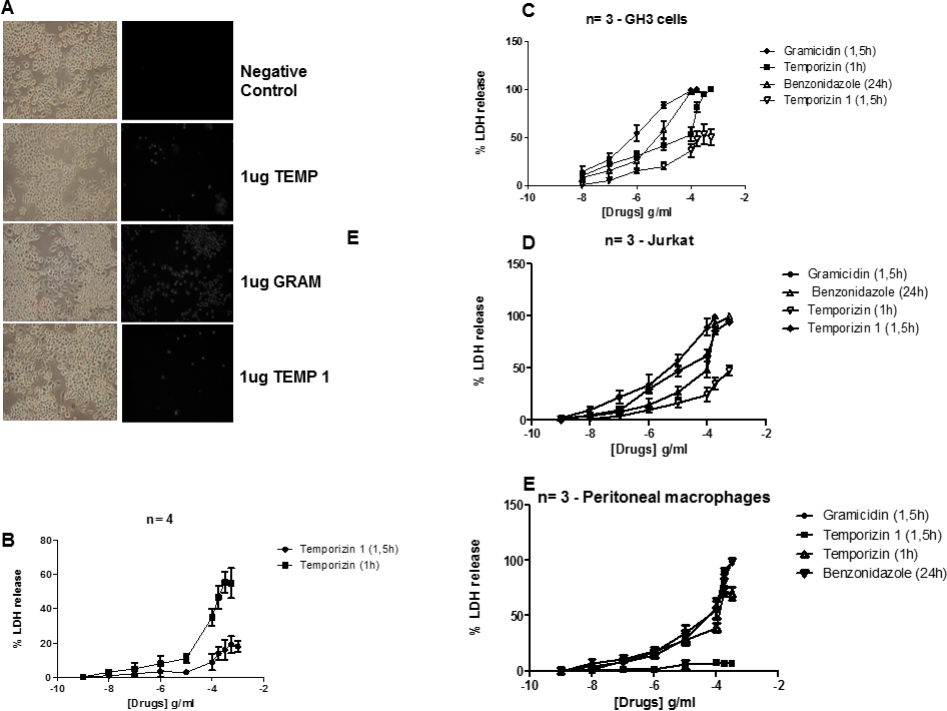
Temporizin-1 possesses low toxicity against mammalian cells of diverse species. A: J774 cells were incubated at 37°C for 60 minutes for a cell permeabilization assay. A single dose of 1 μg/ml was applied to compare the efficiency of the gramicidin (A2), temporizin (A3), gramicidin (A4) and temporizin-1 (A5) peptides to injure the J774 cell line. B: Dose-response relationship of temporizin and temporizin-1 after treatment for 60 and 90 minutes, respectively on mouse J774.G8 cell lineage. The cytotoxic actions was quantified by LDH release assay. C: A graphic representing LDH release performed in human Jurkat cells after adding the peptides and benznidazole at different concentrations. D: A graphic representing LDH release performed in the rat adenoma GH3 cell line after adding the peptides and benznidazole at different concentrations. E: A graphic represention of the LDH Release Assay that was performed after adding the peptides and benznidazole at different concentrations to primary mice peritoneal macrophages The values represent the mean ± SD of three to five experiments (cell permeabilization assay) performed on different days.

As Temporizin-1 displayed cytotoxic effects against trypanosomes with an EC_50_ value similar to temporizin, we investigated whether its action on mammalian cell types caused toxicity or not (Fig 4). We tested temporizin and temporizin-1 effect on a spectrum of cells from distinct species; J774 cell line (which was obtained from reticulum cell sarcoma), mice peritoneal macrophages (a primary mice cell type), human T-cell lymphoblast-like Jurkat cells and rat pituitary adenoma GH3 cells. The action of both peptides was compared to Gramicidin. J774 cells treated with temporizin-1 (1 μg/ml) showed a fewer number of PI labeled cells than temporizin and gramicidin (Fig 4A). The EC_50_ value for LDH release after temporizin-1 application was 129. 3 μg/ml, but the maximal response reached 20%. Temporizin presented an EC_50_ of 83.16 μg/ml with a maximal response in turn of 55% (Fig 4B). Rat GH3 cells were more sensitive to peptide action than J774 cells (Fig 4C). Benzonidazole, the positive control presented an EC_50_ value of 95.72 μM. The values for the peptides were: gramicidin, 1. 2 μg/ml; temporizin, 161.1 μg/ml; and temporizin-1, 341.9 μg/ml (Fig 4C). Among the treatments applied to GH3 cells, only temporizin-1 did not promote a total cytotoxic effect. Human Jurkat cells treated with benzonidazole demonstrated an EC_50_ value of 105.6 μM. The value for gramicidin was 7.5 μg/ml, temporizin was 134.3 μg/ml and temporizin-1 was 59.09 μg/ml. However, temporizin-1 reached a maximal toxic response in 20% of Jurkat cells (Fig 4D). Mice peritoneal macrophages, a primary cell type, was more resistance to all treatments compared to J774, GH3 and Jurkat cells, as expected (Fig 4E). The EC_50_ values were: benzonidazole, 151.8 μM; Gramicidin, 125.5 μg/ml; temporizin, 115 μg/ml; and temporizin-1, 3.6 μg/ml. However, the maximal toxic activity to temporizin was about 75% compared to 7.5% for temporizin-1.

### Trypanocidal action of temporizin and temporizin-1 is associated to cytoplasmic alteration

**Fig 5:**
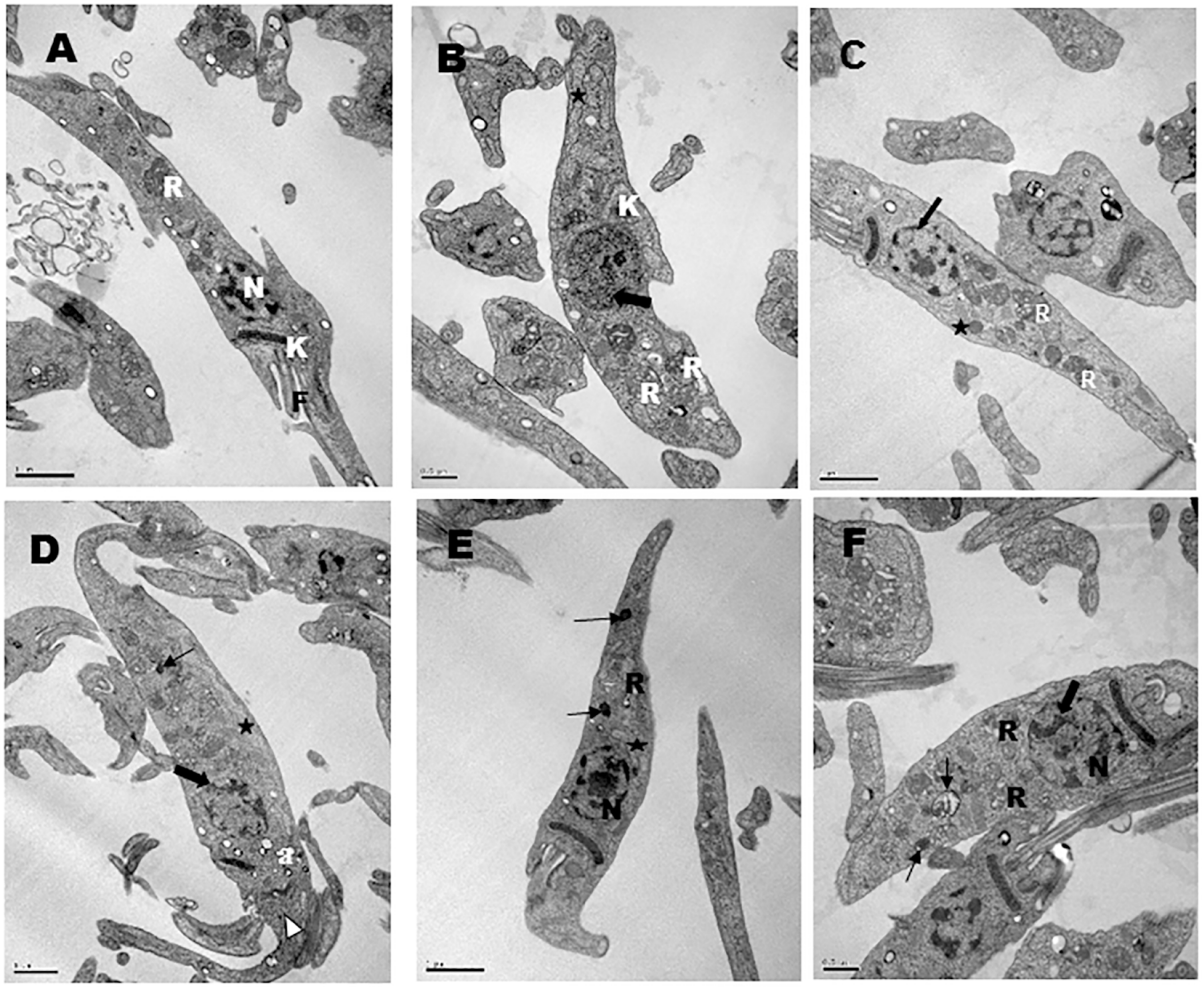
Transmission electron microscopy of *Trypanosoma cruzi* epimastigotes forms treated for different times with 1μg/ml of temporizin (TZ) or temporizin-1 (TZ1) or Gramicidin (Gra). (A) Untreated parasite, showing the characteristic structure of kinetoplast (K), reservosome (R), flagellum (F) and nucleus (N); (B) Parasites treated for 30 minutes with TZ showing abnormal chromatin condensation (large arrow); mitochondrial cristae disorder (star); kinetoplast disorganization (K) and swelled reservosome (**R**); (C) 90 minutes of treatment with TZ1 showed mitochondrial cristae alteration (star); disorganization in the reservosome morphology (**R**); chromatin reduction (large arrow); (D) 3 hours of TZ1 treatment showing chromatin condensation reduction (large arrow); reservosome degeneration (thin arrow); concentration of acidocalcisomes in the anterior region (**a**); microvesicles releasing near Golgi (white arrow head); mitochondrial cristae reduction (star); (E) 90 minutes of Gra treatment showing a reduction of the mitochondrial cristae (star), disorganization of reservosome morphology (R) and reservosome degeneration (thin arrow); (F) 3 hours of Gra treatment showing chromatin alteration (large arrow), degeneration of reservosomes (thin arrow) and alteration in the reservosome morphology (R).

Untreated parasites did not exhibit structural membrane or intracellular alteration (Fig 5A). Temporizin treatment for 30 minutes induced chromatin condensation (large arrow), mitochondrial cristae disorder (star), kinetoplast disorganization (K) and reservosome swelling (R) (Fig 5B). No alteration to the plasma membrane was seen. Temporizin-1 evoked a prominent trypanocidal effect similar to temporizin after 90 min of exposure (Fig 5C). There was mitochondrial cristae alteration, disorganization in the reservosome morphology and chromatin condensation. Temporizin-1 treatment for 3h exhibited chromatin condensation reduction, reservosome degeneration (thin arrow), microvesicle releasing near Golgi (white arrow head) and mitochondrial cristae reduction (Fig 5D). In the Fig 5E, gramicidin treatment for 90 minutes reduced mitochondrial cristae, it induced disorganization of the reservosome morphology and degeneration. The treatment for 3h altered chromatin condensation and affected reservosomes (Fig 5F). No peptides altered plasma membrane morphology until treatment for 3h.

### Temporizin induce ionic currents in mammalian cell membrane

**Fig 6:**
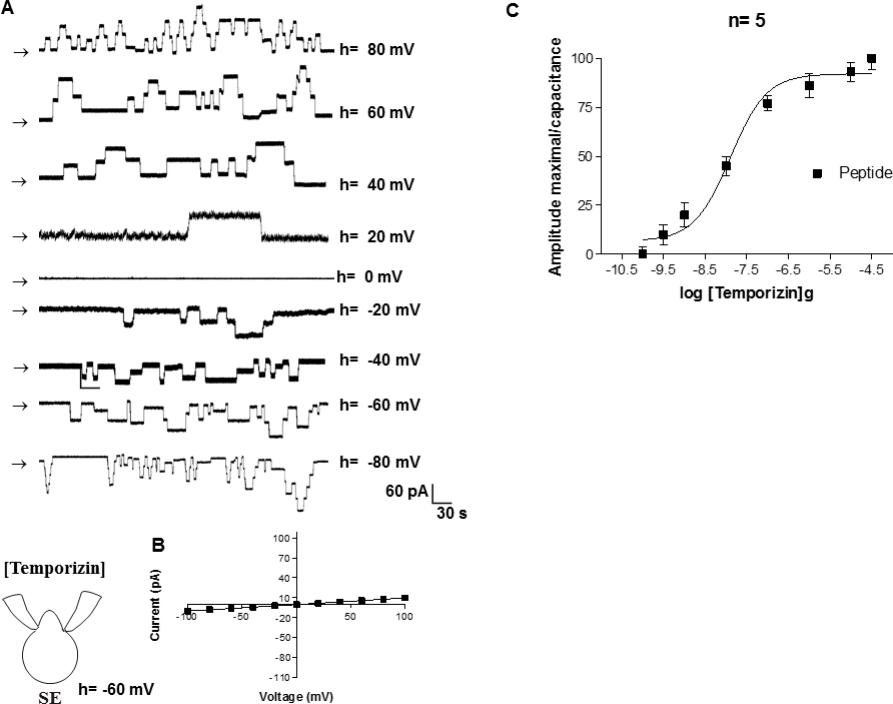
Temporizin induces a macroscopic ionic current in the HEK-293 mammalian cell line. A: Macroscopic ionic currents of a whole-cell patch from HEK-293 cells that were induced by different temporizin concentrations. The ionic currents (far right) elicited by temporizin are indicated by vertical arrows. The holding potential ranged from +100 mv to -100 mV. B: The mean I-V relations for the whole-cell configuration. C: Whole-cell recordings obtained from HEK-293 cells as a function of varied temporizin concentrations. The values represent the means ± SD of six experiments performed on different days.

A possible mechanism for temporizin peptide to generate toxicity in wild-type mammalian cells could be formation of ionic channels. In this case, as the peptide induced a noticeable LDH release in distinct cell lineages, the hypothetical ionic channel cut off could support the passage of other substances with therapeutic potential. We investigated this possible mechanism of action of the temporizin using electrophysiology of whole-cells and in a cell-attached configuration. Macroscopic currents recorded by whole-cell configuration were observed after the application of 1 μg/ml temporizin in J774 cells. We applied holding potentials from +80 mV to -80 mV in the bath. As can be observed at Fig 6, temporizin-mediated macroscopic ionic currents did not exhibit voltage dependence (Fig 6A and 6B). In addition, this macroscopic ionic current was dependent on temporizin concentration with EC_50_ value of 50.53 ng/ml (Fig 6C). Single channel recordings of the temporizin showed activity in positive and negative potentials. Temporizin channel unitary conductance was 970 pS in 60 mV. Two or more channels in the same recording were observed in positive and negative holdings, although an augmented open frequency is recorded in more elevate holding potential (Fig 6A and 6B). In comparison to whole cell, cell attached experiments detected channel activity with inferior temporizin concentrations, EC_50_ = 12.68 ng/ml (Fig 6C).

### Temporizin-1 peptide forms an ionic channel minor than temporizin in mammalian cells

**Fig 7:**
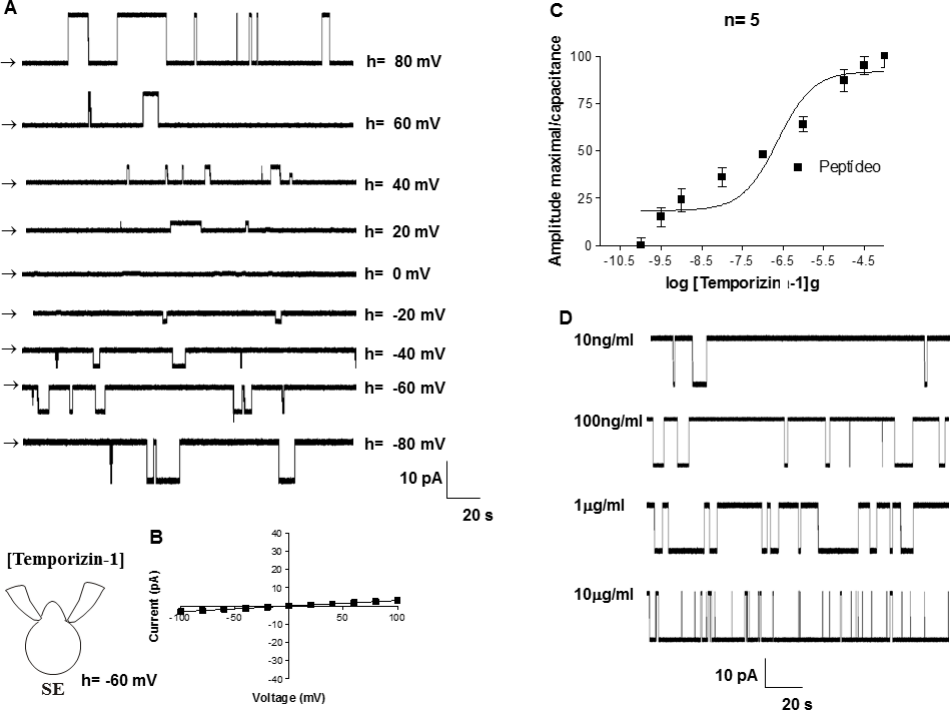
Temporizin-1 induces a macroscopic ionic current in the HEK-293 mammalian cell line. A: Macroscopic ionic currents of a whole-cell patch from HEK-293 cells induced by different temporizin-1 concentrations. The ionic currents (far right) elicited by temporizin-1 are indicated by vertical arrows. The holding potential ranged from +100 mv to -100 mV. B: The mean I-V relations for the whole-cell configuration. C: Whole-cell recordings obtained from HEK-293 cells as a function of the varied temporizin-1 concentrations. D: Single channel recordings at distinct temporizin-1 concentrations. The values represent the means ± SD of six experiments performed on different days.

Using whole-cell configuration, 1 μg/ml temporizin-1 was added in the bath (Fig 7). As represented in the Fig 7A, there was no voltage dependence and the reversion potential was in turn of 0 mV (Fig 7B) that gave an EC_50_ value for temporizin- 1 to form ionic channels of 23.03 μg/ml (Fig 7C). The unitary conductance measured was of 130 pS.

## Discussion

Current treatments for trypanosome infections in chagasic patients rely on the drugs benznidazole and nifurtimox. These drugs are the only ones with proven efficacy against Chagas disease. However, they are limited due to their high toxicity and they have unproven benefits during the chronic phase of the disease [45]. Further difficulties include an absence of effective vaccines and prophylactic measures, plus there is an increasing presence of drug resistance in the trypanosomatid population [46, 47]. These restrictions encourage the search for new anti-parasitic effectors that have a broad spectrum of activity, low toxicity in the recipient and a low propensity to induce microbial resistance. Antimicrobial proteins or peptides (AMPs) possess many of these properties, and were chosen for use in our model.

Frogs are naturally refractory to *Trypanosoma cruzi* [31], and amphibian skin is the richest source of natural peptides [28, 30]. The *Rana* genus has a global distribution with approximately 250 different species [34]. One species, the European red frog *Rana temporaria* produces temporin peptides, which possess properties unique to the Ranidae family due to the absence of the “Rana box” motif and they are amidated at their C-termini [48]. In addition, temporins are the smallest amphipathic α-helical containing AMPs found in nature (10–14 amino acids) with a net positive charge at a neutral pH ranging from 0 to +3. They can act against a wide range of pathogens and have low toxicity in mammalian cells. Their mode of action includes the perturbation of the pathogen plasma membrane, which allows the passage of small and large molecules in a rapid and dose-dependent manner [29].

Previous studies on peptides found in the secretions of 8 species of anurans and 1 specie of gymnophionan, showed activity against protozoan parasites, which are the etiological agents of visceral leishmaniasis, Chagas disease and toxoplasmosis [49]. Specifically for temporin peptides acting on trypanosomes, Temporin-SHd (APD ID: AP02118) isolated from the North-African frog *Pelophylax saharicus* gave significant inhibitory effect against *T*. *brucei* and *T*. *cruzi* [50, 51]. The EC_50_ value obtained was 16.8 μM to trypanocidal action against *T*. *cruzi* and mammalian cells toxicity range from 25 μM to human erythrocytes and 594 μM to human HepG2 cell lineage. To obtain a peptide with the ability to form gramicidin pores with low toxicity in mammalian cells, we developed a hybrid peptide, temporizin, based on the sequence responsible for temporin A insertion into the cell membrane, anchored to a poly-Leu peptide sequence for stability and combined with the sequence responsible for the ionic channel formation of gramicidin.

After a 1h treatment with temporizin, the peptide promoted a dose-dependent trypanocidal effect (Fig 1), but it was moderately toxic to mammalian cell type (Fig 2). The calculated EC_50_ value of the temporizin trypanocidal action was 795 ng/ml and CC_50_ value was 83.16 μg/ml in J774.G8 cells. To minimize toxicity towards mammalian cells, the temporizin sequence was modified to generate temporizin-1. The new peptide displayed a similar trypanocidal activity with an EC_50_ of 887.2 ng/ml, but the toxicity for mammalians cells was reduced with a CC_50_ = 129.3 μg/ml that had a maximal LDH release in a physiological range (Fig 4).

Previous studies on isolated peptides from the cutaneous secretion of the tree frog *Phyllomedusa nordestina* reported trypanocidal activities for dermaseptin 1, dermaseptin 4, phylloseptin 7 and phyllospetin 8 as EC_50_ values of 1.64, 0.86, 0.69 and 0.94 μg/ ml, respectively [52]. Their cytotoxity against wild-type mammalian cells was determined as CC_50_ values of > 48.2 μg/ml, >34.6 μg/ml, 34,42 μg/ml and >20,471 μg/ml, respectively [52]. In comparison to the hybrid peptides presented here, their trypanocidal values were ~10 fold inferior and their toxicity in mammalian cells was greater. Another peptide, mellitin (MW 2846.46), was tested against the three *T*. *cruzi* developmental forms (epimastigotes, trypomastigotes and amastigotes). Treatment of epimastigotes for 48h yielded an EC_50_ of 2.44 μg/ml and after 96h, 4.51 μg/ml. The cytotoxic action on mammalian LLC-MK2 cells produced a CC_50_> 5 μg/ml [53]. In comparison to our results, although trypanocidal activity was compatible, this peptide exhibited greater toxicity in mammalians cells than temporizin or temporizin-1. Further, the antimicrobial peptides apidaecin, melittin, penaeidin, cecropin A, moricin and magainin 2 were tested against *T*. *cruzi*. Of these, only moricin did not exhibit a trypanosomal effect [51, 54]. The EC_50_ values to peptides effect were 320.3 μg/ml, 81.4 μg/ml, 419.57 μg/ml and 85.39 μg/ml, respectively. All of their EC_50_ values were more than 100 times superior to temporizin-1.

For both temporizin and temporizin-1, we performed an electron microscopic examination of the treated trypanosomes to determine the cytoplasmic effects. Following 30 minutes with temporizin and 90 or 180 min with temporizin-1, intracellular destruction was observable. Electron micrographs displayed alterations to mitochondria and nuclear DNA. In some case, there was increase in the number of reservosomes suggesting drug accumulation (Fig 4). Interestingly, the plasma membrane was not alter for any peptide exposure.

For mellitin, its actions on *T*. *cruzi* epimastigotes forms appears to promote autophagy death, but appears as apoptosis in trypomastigote that suggests a selective mechanism of action depending on parasite form [53]. In all cases, mellitin seemed to not affect trypanosome plasma membrane. This is in contrast to the activity of human defensin-1α, which exhibits a trypanocidal function against the trypomastigote and amastigote forms through a toxicity that is mediated by the formation of membrane pores with a subsequent induction of DNA fragmentation [55]. For the peptides dermaseptin 4, dermaseptin 7, phylloseptin 7 and phylloseptin 8, their mode of action alters the plasma membrane fluidity of *T*. *cruzi* based on Sytox Green uptake over 60 minutes [52]. Since the plasma membrane was not grossly altered by temporizin and temporizin-1 according to electron microscopy, yet showed an uptake of PI, we suggest that these peptides also change the fluidity of *T*. *cruzi* plasma membrane.

The toxicity of temporizin to mammalian cells, which was constructed with the pore forming region of gramicidin, it possibly forms pores in membranes of microorganisms and mammalian cells [56]. Electrophysiology was used to test the hypothesis that temporizin may form ionic channels in mammalian plasma membrane (Fig 6). Temporizin was shown to form voltage-insensitive ionic channels with a unitary conductance of 970 pS. Temporizin-1 was also tested for its ability to form ionic channels (Fig 7). The unitary conductance of the channel recorded, 130 pS, was lower than temporizin, as expected since the difference between the peptides was a reduction in the region from gramicidin.

The observed difference in unitary conductance may be directly related to mammalian cell injury as quantified by LDH release. Several groups have shown that the mechanism of AMPs effects is mediated by forming ionic channels in planar lipid bilayers, although not in neutral membranes [57–63]. In some cases, the peptide permeabilizes planar lipid bilayer membranes at concentrations similar to those observed to kill mammalian cells *in vitro* [64–66]. In contrast, there is a reduced number of results about direct antimicrobial peptide pore forming in native plasma membranes. The chimeric peptide Cm_18_-Tat_11_, composed of the Tat_11_ arginine-rich motif (YGRKKRRQRRR) [67] fused to CM_18_, (KWKLFKKIGAVLKVLTTG) [68, 69], promotes an irreversible pore forming activity at CHO-K1 cells. The recorded voltage-insensitive channel displayed an ionic channel with single conductance comparable to temporizin channel. The channels recorded by temporizin-1 showed similarities to the peptide Alameticin that induced an ionic channel that was active in positive and negative holding potentials with an approximate unitary conductance of 58 pS [70].

The conversion of temporizin to temporizin-1, created by shortening the four residues related to the gramicidin ionic channel pore, appeared to have generated a preferential activity towards trypanosomes compared to mammalian cells, which could relate to the number of ionic channels formed and their ionic current amplitude. The number of ionic channels formed by temporizin-1 in the patch, when using the attached cell configuration, was decreased in comparison with that of temporizin. This property seems to interfere in the mammalian cell toxicity, because temporizin-1 has a lower toxicity than temporizin. Whereas, the conductance of the temporin-1 channels are lower than that of the temporizin channel conductance, which may explain the slight reduction in the *T*. *cruzi* toxicity observed between them. Interestingly, both temporizin-1 and temporizin exhibited low toxicity towards primary cells than neoplastic cell lines suggesting that these peptides could be used in the treatment of neoplasms *in vivo*. Previous publications have shown the utility of antimicrobial peptides in the treatment of tumors [71, 72].

Temporizin-1 appears to form ionic channels in mammalian cell membranes, which most likely underlies its toxicity. However, its toxicity towards trypanosomes appears to be attributable to an intracellular affect rather than pore formation. This difference in action may be result of the distinct constituents determined for the plasma membrane of mammalian and trypaonsome plasma membranes that show differential quantities of total phospholipids and sterol [73–76]. Therefore, the peptide may find elements to anchor and insert in the membrane of mammalian cells. In contrast, while the peptide may anchor to the protozoa membrane, it passes directly to the cytoplasm where it alters organelles rather than remaining in the plasma membrane as a pore.

In conclusion, we have shown that the antitrypanosomal activity of the naturally occurring temporin A and gramicidin peptides could be enhanced by their synthetic fusion that, together with poly-leu section, formed the hybrid peptide temporizin. Its characterization showed a moderate toxicity towards mammalian cells, which prompted a reduction in its amino acid residues to make the temporizin-1 peptide. Temporizin-1 presented an improved anti-trypanosomal activity compared to temporizin and gramicidin as well as a lower toxicity in mammalian cells. The data suggest that temporizin-1 has characteristic desired for a good candidate in the development of novel anti-trypanosomal drugs.
